# First Report of Influenza D Virus in Dairy Cattle in Pakistan

**DOI:** 10.3390/v16121865

**Published:** 2024-11-29

**Authors:** Sajid Umar, Aftab Ahmed, Sajjad Hussain Gulraiz, Shaban Muhammad, Jieshi Yu, Arslan Rasool, Renata Koviazina, Aysun Yilmaz, Huseyin Yilmaz, Benjamin D. Anderson

**Affiliations:** 1 Global Health Research Center (GHRC), Duke Kunshan University, Suzhou 215316, China; 2 Division of Natural & Applied Sciences (DNAS), Duke Kunshan University, Suzhou 215316, China; 3 Friesland Campina Engro Pakistan Ltd., Karachi 75600, Pakistan; 4 Punjab Agriculture and Meat Company (PAMCO), Lahore 54000, Pakistan; 5 State Key Laboratory of Swine and Poultry Breeding Industry, Agro-Biological Gene Research Center, Guangdong Academy of Agricultural Sciences, Guangzhou 510640, China; jieshi_yu@outlook.com; 6 College of Food Science and Technology, Huazhong Agricultural University, Wuhan 430070, China; 7 Department of Virology, Veterinary Faculty, Istanbul University-Cerrahpasa, 35500 Istanbul, Büyükcekmece, Türkiye; 8 Department of Environmental and Global Health College of Public Health and Health Professions, University of Florida, Gainesville, FL 32610, USA

**Keywords:** influenza D virus, cattle, RT-PCR, phylogeny, Pakistan

## Abstract

Influenza D virus (IDV) is a newly emerged zoonotic virus increasingly reported worldwide. Cattle are considered the main reservoir of IDV, although it was first isolated from pigs. IDV infects multiple animal species and contributes to the bovine respiratory disease complex (BRDC). To date, there has been no report on the presence and frequency of IDV among cattle herds in Pakistan. In this study, we collected nasal swabs from cattle and performed virological surveillance of IDV via qRT-PCR. Among 376 swab samples, IDV was detected in 9 samples (2.4%). Four dairy cattle farms were positive for IDV; two IDV-positive samples (two/nine, 22.2%) belonged to asymptomatic cattle, while seven IDV-positive samples (seven/nine, 77.8%) were from cattle showing respiratory clinical signs, including two with a recent history of abortion and mastitis. Partial sequences of the hemagglutinin–esterase-fusion gene of IDV were obtained from nine qRT-PCR-positive samples. Notably, all IDV strains in this study clustered within the D/OK lineages in phylogenetic analysis. A 98.8–99.6% genetic identity to its European and US counterparts indicates that the IDVs are closely related. The D/OK lineage of IDV was previously unreported in Pakistan. This is the first report of IDV in Pakistan. We confirmed that IDV is circulating among cattle herds in Pakistan. This study underscores the importance of virological surveillance to monitor the ecology of IDV for better animal and public health. The continued spread of IDV and its adaptation to various hosts necessitate further epidemiological studies.

## 1. Introduction

Influenza D virus (IDV) is a newly discovered virus with zoonotic potential belonging to the family *Orthomyxoviridae* (a family that includes the four types of influenza viruses). The virus was first isolated from pigs in 2011, but retrospective serologic studies on bovine serum samples reveal that the virus has been circulating in cattle since at least 2003 [1,2]. Cattle are the most probable main source of viral transmission [1,2,3,4]. Over the past decade, IDV has been reported worldwide, with cases in the Americas, Asia, Europe, Australia, and Africa [1,3,5,6,7,8,9,10,11]. It has been associated with mild-to-moderate respiratory disease in cattle and can spread through direct contact or short-distance aerosol transmission. IDV has been detected in both clinically sick and asymptomatic cattle in the past. However, higher detection rates have been linked to the presence of cattle respiratory infections. IDV is now recognized as a significant contributor to the development of bovine respiratory disease complex (BRDC), leading to substantial economic losses in the global dairy industry [2,3,6,12,13,14,15]. Additionally, it is predicted that IDV suppresses the innate immune responses in cattle against bacteria, thereby increasing their susceptibility to secondary bacterial infections [16].

Since 2011, evidence of active IDV infections (virological detection) or pervious exposure (antibody detection) have been reported in several animal species, including horses, dogs, cattle, pigs, sheep, camels, and goats. Recent bioaerosol evidence suggests that chickens can also be infected with this virus [1,4,16,17,18,19,20,21,22]. Under experimental conditions, IDV can infect several animal species, including mice, guinea pigs, cattle, pigs, and ferrets [23,24,25]. In human cells (A549 and HRT-18G cell lines) and animal cells (MDCK, ST, MARC-145 cell lines), IDV infection has also been observed [2,23,26,27]. The zoonotic potential of IDV is demonstrated by the ability of the virus to spread in various animal species and cell lines. While this has not yet been fully clarified, serologic and virologic testing suggest that IDV may infect humans, especially those with professional exposure to animals, such as farm workers and veterinarians [22,28,29,30,31].

IDVs are enveloped, spherical to polymorphic viruses, measuring approximately 100–120 nm in diameter. Viruses of types C and D possess a unique surface glycoprotein, the hemagglutinin–esterase fusion protein (HEF), while types A and B have two main surface glycoproteins: hemagglutinin (HA) and neuraminidase (NA). Like influenza C viruses (ICVs), the genome of IDVs consists of seven RNA fragments. To date, IDV can be classified into six lineages based on the HEF gene: D/OK (D/swine/Oklahoma/1334/2011); D/Bursa2013 (D/bovine/Turkey-Bursa/ET-130/2013); D/660 (D/bovine/Oklahoma/660/2013); D/Yama2016 (D/bovine/Yamagata/10710/2016); and D/Yama2019 (D/bovine/Yamagata/1/2019). The HEF gene is the primary target of neutralizing antibodies produced during IDV infection [3,11,32]. The D/OK and D/660 lineages have been detected more frequently in North America, Europe, and China [24], while the D/Yama2019 lineage has primarily been found in Japan and China. A new D/CA2019 lineage has recently been identified in California, Brazil, and Turkey [1].

The first case of IDV from Asia was detected among healthy cattle in Shandong, China, in 2014 [33]. Since then, several studies have shown the circulation of IDV among cattle in various provinces of China [6,34,35]. The first case of IDV in cattle in Japan was reported in 2016 in Japan’s Ibaraki Prefecture [36]. More recently, in 2023, IDV infections were reported in cattle and pigs in the Republic of Korea as well [11].

Continued reports of IDV detections in multiple countries reinforce the idea that the virus is spreading globally. Its infectivity and cross-species transmissibility make IDV an emerging epidemiological threat that necessitates ongoing surveillance and further research. Despite the growing evidence, no epidemiological survey has been conducted on IDV in dairy cattle in Pakistan, even though it plays a pivotal role in the country’s economy. Over the past eight years, Pakistan has witnessed a shift towards commercial and corporate dairy farming, with several hundred new dairy farms opening during this period. Most of these farms imported cattle breeds, such as Holstein Friesian and Jersey, from Australia and the United States. With increasing reports of IDV infections among cattle globally, including in nearby China, we conducted a study to examine the prevalence of IDV in cattle in Punjab, Pakistan. This work will help determine whether IDV contributes to BRDC in Pakistani cattle.

## 2. Materials and Methods

**Ethics statement:** This study was conducted in accordance with the guidelines set by the World Organization for Animal Health (WOAH) [25]. Since only nasal swab samples were collected, ethical approval from the Institutional Animal Care and Use Committee (IACUC) was not required for this non-invasive study.

**Figure 1:**
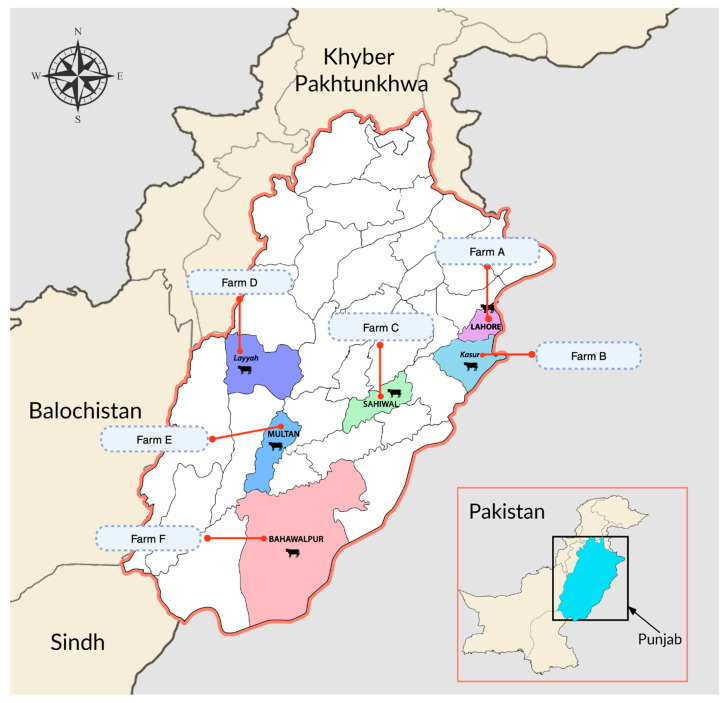
Farm locations for study of influenza D virus among dairy cattle in Punjab province, Pakistan.

**Sampling strategy:** As part of virological surveillance, we collected nasal swab samples (n = 376) from six dairy farms across various districts of Punjab (Lahore, Kasur, Sahiwal, Multan, Lehigh, Bahawalpur) between January 2023 and March 2024 (Figure 1). Nasal swabs were collected randomly from adult cows, heifers, and sick animals. Adult cows included both lactating and non-lactating (dry) cows. Samples were collected only from animals aged between 1 and 8 years; calves were not sampled in this study. Data on farm locations, farm type, animal type, sample type, health status, and detection rates were also recorded (Table 1). Four farms (A, B, C, and E) were modern commercial dairy farms with more than 1000 animals, where animals were kept in captivity with appropriate biosecurity measures. Animals at these modern commercial dairy farms were fed silage, hay, and grain concentrates. There were two small conventional dairy farms (D and F) with fewer than 500 animals. These conventional small-scale dairy farms were located in Layyah and Bahawalpur and did not follow appropriate biosecurity measures. The cattle at conventional dairy farms were mainly fed fodder crops (alfalfa, sorghum, maize, and oats) and grain concentrates.

**RNA isolation:** Swabs were subjected to RNA isolation using a commercial RNA extraction kit (Cat#9766, Takara, Dalian, China). To ensure the integrity of the reagents and the extraction process, both positive and negative controls were included. Nucleic acids were eluted using 60 μL of elution buffer. After extraction, the nucleic acid samples were stored at −80 °C for future analysis.

**Virological screening:** Extracted samples were screened for IDV-RNA using a one-step real-time RT-PCR kit (HiScript III One Step qRT-PCR Probe Kit, Vazyme, Nanjing, China) following the manufacturer’s instructions. Specific primers and probes were used to target the polymerase basic 1 (PB1) gene, as described previously [5]. After the initial reverse transcription step at 55 °C for 15 min, the targeted gene was amplified under the following conditions: 95 °C for 3 min, followed by 45 cycles of 95 °C for 10 s, and 60 °C for 30 s. Negative and positive controls were added in the PCR reactions. Samples with a Ct value below 38 were considered positive for IDV-RNA.

**Genetic analysis:** Samples that tested positive via qRT-PCR were further analyzed using conventional RT-PCR to determine the genetic lineage of IDV, as previously described [26]. A pair of primers (HEF-F: AACCRCATCTTCTTGTTCTTCA and HEF-R: TGCTTCTTCWGTGGCATTATCT) were used to amplify a region of the HEF gene (position 582-1077) of IDV. A 50 μL master mix was prepared using the HiScript II One-Step RT-PCR Kit (Vazyme, Nanjing, China), consisting of 1 μL of each primer (10 μM), 25 μL of 2× One Step Mix (Dye Plus), 2.5 μL of One-Step Enzyme Mix (including PrimeScript RTase, DNA polymerase, and RNase inhibitor), 15.5 μL of ultrapure water, and 5 μL of viral RNA. We prepared the master mix in a C1000 Touch™ Thermal Cycler (Bio-Rad Laboratories, Inc., Hercules, CA, USA) using the following thermal cycling conditions: 5 min at 95 °C, followed by 40 cycles of 30 s at 95 °C, 30 s at 55 °C, and 30 s at 72 °C, with a final 5 min extension at 72 °C. PCR amplicons were analyzed on a 2% agarose gel using a GenoSens 1880 Gel Imaging Analysis System (GenoSens 1880, Clinx, Shanghai, China). A 496 bp fragment of the HEF gene was sequenced by commercial sequencing company (GENEWIZ, Suzhou, China). The partial HEF gene sequences were deposited in GenBank (accession numbers: PP763073-PP763081).

**Phylogenetic analysis:** Reference IDV sequences (D/swine/Oklahoma/1334/2011 (D/OK); D/bovine/Oklahoma/660/2013 (D/660); D/bovine/Yamagata/10710/2016 (D/Yama2016); D/bovine/Yamagata/1/2019 (D/Yama2019); California/0894/2019 (D/CA2019); and Bursa/ET-130/2013 (D/Bursa 2013) were retrieved from the NCBI GenBank database. Raw sequences were edited and aligned using the ClustalW 2.1 alignment tool in BioEdit software (Ibis Biosciences, Carlsbad, CA, USA). Pairwise matching was performed to calculate the nucleotide identity between the IDV strains in this study and the reference strains. A phylogenic tree was constructed using the neighbor-joining method with 1000 bootstrap replicates and the maximum composite likelihood method [37], implemented in MEGA11 software (http://www.megasoftware.net) [27]. The number of substitutions per site was indicated by a scale bar. A total of 63 nucleotide sequences of HEF gene were used in the phylogenetic tree analysis.

## 3. Results

### 3.1. Clinical Findings and Frequency of IDV

Among the 376 samples, 9 samples (9/376, 2.4%) were found positive for IDV-RNA. As described in Table 1, the positive rate (%) for IDV was the highest in the Lahore district (4/59, 6.8%), followed by Kasur (2/73, 2.7%), Multan (2/77, 2.6%), and Layyah (1/65, 1.5%). IDV was not detected in the samples collected from the Sahiwal and Bahawalpur district (Farm C and Farm F). Two IDV-RNA-positive samples (two/nine, 22.2%) belonged to asymptomatic cattle, while seven IDV-RNA positive samples (seven/nine, 77.8%) belonged to cattle showing respiratory clinical signs, including two cattle with a recent history of abortion and mastitis. Our preliminary statistical analysis showed that the IDV detection rate was significantly higher in sick animals than in healthy animals (*p* < 0.05). Four dairy cattle farms were positive for IDV-RNA, which were in Lahore, Kasur, Layyah, and Multan. IDV-RNA was not detected from dairy farms in the Sahiwal and Bahawalpur districts. The other bovine respiratory pathogens which are frequently involved in the bovine respiratory complex were not tested in this study (e.g., bovine coronavirus, bovine parainfluenza type 3 virus, bovine syncytial respiratory virus, bovine herpesvirus type 1, *Mannheimia haemolytica*, *Mycoplasma bovis*, *Pasteurella multocida*, and *Histophilus somni*). However, we screened these samples for avian influenza virus (H5N1), and no evidence for H5N1 infection was found [28].

### 3.2. Phylogenetic Analysis and Nucleotide Homology of HEF Gene

**Figure 2:**
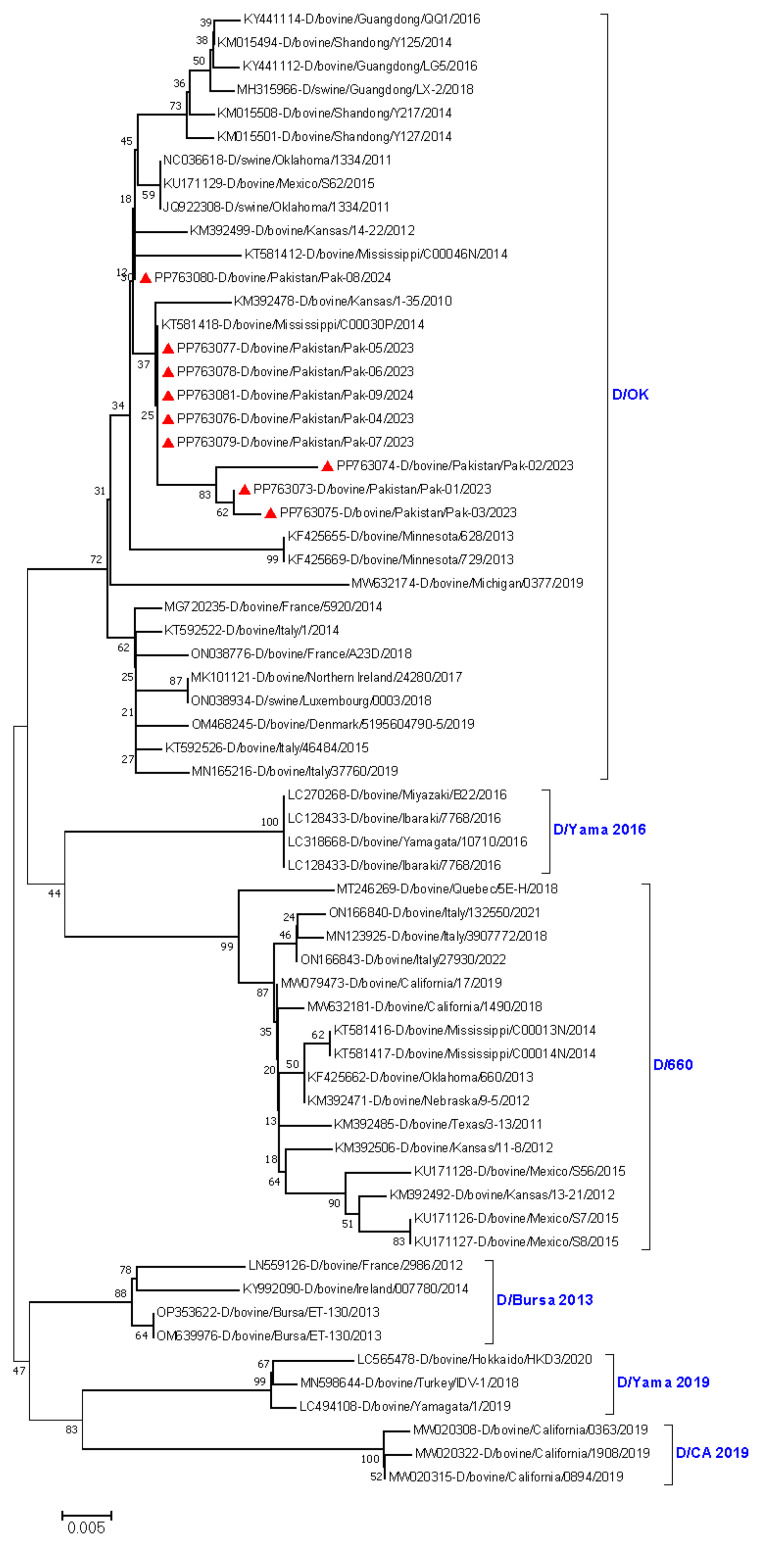
Partial hemagglutinin–esterase-fusion gene (496 bp)-based phylogenetic tree of influenza D viruses among dairy cattle in Punjab province, Pakistan. The IDV sequences of the present study have been highlighted with red tringles. PP763073, PP763074, PP763075, and PP763076 were from the same farm (Farm A); PP763077 and PP763078 were from the same farm (Farm B); PP763079 was from farm D; and PP763080 and PP763081 were from same farm (Farm E). No sample was found positive for IDV at Farm C or Farm E.

Preliminary analysis of HEF partial gene (496 bp) indicated D/OK lineage circulation in Pakistan because all IDV strains in this study clustered with D/OK lineages in phylogenetic analysis. A nucleotide identity of 99–99.8% was observed among the sequences of the nine IDV strains detected in the present study. The sequence analyses revealed genetic identity ranging from 98.8% to 99.6% between the nine IDV strains of this study and the reference strains from the United States (D/bovine/Mississippi/C00030P/2014; D/bovine/Kansas/14-22/2012) and China (D/bovine/Guangdong/SQ/2018; D/bovine/Shandong/Y125/2014). The IDV strains in the present study clustered closely with each other in the phylogenetic tree, revealing low evolutionary distance between them. The IDV strains clustered closely with the D/OK lineage reference strains from China and the USA. Interestingly, PP763073, PP763074, and PP763075 formed a separate cluster within the D/OK lineage (Figure 2).

## 4. Discussion

Influenza D virus (IDV) is a virus that has been increasingly recognized worldwide, and multiple animal species, including goats, sheep, camels, horses, buffalo, and deer, have been found positive for this virus [1,3,4,6,18,19]. IDV has a wide geographical range, and, to date, it has been confirmed molecularly or serologically in more than 30 countries [1,3,6]. In this study, molecular screening was performed to detect IDV RNA from samples collected from cattle belonging to farms in Punjab province. This is the first report on IDV from Pakistan; therefore, a direct comparison between findings could not be made.

In the present study, IDV was detected in cattle in Pakistan but, overall, at a low prevalence (2.4%) (Table 1). IDV-positive rates were 1.5−6.8% among different cattle farms. This low prevalence might be attributed to good management practices at farms, low virus circulation, and the low number of samples in this study. Over the years, a variable pattern of IDV prevalence has been reported worldwide. In a recent study in China, IDV RNA was detected in 51 (11.1%) samples collected from cattle from different provinces. IDV RNA detection rates varied from 4.7% to 31.3% among the provinces [6]. In addition, IDV-positive rates of 12.8% (20/156), 4% (10/250), and 0.66% (3/453) were reported by Zhai et al. [26], Yu et al. [29], and Jiang et al. [30], respectively, from China. From 2013 to 2014 in the United States, IDV was detected in 4.8~18.0% of cattle. However, IDV was only detected in 0.07% of cattle in 2016 in the United States [3,11,31,32]. A positivity rate of 1.3~44.1% in cattle was recorded in Europe during the period 2010–2020 [24]. Furthermore, the IDV-positive rate in Japan was 2.1% (8/377) in 2016 [33]. In a recent study from the Republic of Korea, 1.4% of cattle were found positive for IDV [11]. Epidemiological studies have shown a higher prevalence in cattle compared to other species [1,3,4,11]. During this study, a higher detection rate for IDV was noticed in farms located in central Punjab (Lahore, Kasur: six/nine, 66.6%) than in farms located in Southern Punjab (Layyah, Multan: three/nine, 33.3%). IDV was not detected in two farms in Punjab (Farm C and Farm F). It is speculated that IDV may not have been introduced into these farms, leading to negative detection in this study. Alternatively, it is suggested that the distribution of IDV among cattle farms in Punjab province is likely not homogeneous. Good farm management, including stringent biosecurity and less animal movement between farms, could lower the prevalence of IDV. Therefore, it is reasonable to expect that the prevalence of IDV would vary from one farm to another. However, due to the small number of samples, we cannot conclude with confidence that IDV is absent at some farms. Sample size is an important aspect of epidemiological surveillance and disease monitoring, which cannot be ignored. A small sample size can limit the statistical power of a study, making it challenging to draw confident conclusions about the general population from the data collected. Therefore, we urge continuous monitoring with extensive sampling to ensure that IDV is truly not present at these farms. Alternatively, IDV-RNA-negative farms can be analyzed for antibodies to IDV via an ELISA or HI test to investigate IDV exposure in the past.

A total of eight IDV-positive samples belonged to imported cattle breeds. These farms import cattle from other countries. Moreover, only one sample was found positive in cross-bred indigenous cattle breeds in Layyah; this farm had no history of animal importation from other farms or countries. Mild-to-moderate respiratory clinical signs were observed in seven IDV-positive cattle at three farms (Farm A, D, E); however, two IDV-positive cattle at Farm B did not show clinical signs. No mortality was reported among IDV-positive cattle in this study. The IDV detection rate was significantly higher (seven/nine, 77.8%) in diseased animals than in asymptomatic animals (two/nine, 22.2%) in this study. This finding suggests a potential contribution of IDV to respiratory infection. It has been reported that IDV causes mild respiratory disease in cattle and could play a role in developing the bovine respiratory disease complex [1,3,6,11,26,34]. IDV seropositivity increases with age, possibly due to the horizontal spread of IDV within a herd [10]. On the contrary, the qPCR-positivity rate is higher in younger than in older animals. Over the years, higher positive rates for IDV have been observed in cattle suffering from respiratory infection [1,3,6,12,13,14,26,35]. A wide range of IDV positivity (1.3–29.1%) has been previously reported in cattle with respiratory infections [5,7,34,35,36]. A lower prevalence of IDV infection (0.6–2.4%) has been observed in healthy cattle [30,36]. The detection of IDV in seemingly healthy cattle populations indicated that the virus could circulate silently within herds without causing overt illness, complicating surveillance and control measures. Therefore, antibodies to IDV need to be investigated in future studies.

The genome analysis revealed the circulation of one genetic lineage (lineage C/OK) in the cattle population of Pakistan. It seems that the D/OK lineage of IDV is the only prevalent lineage in Pakistan. Our study supports the previous observations that D/OK lineage is circulating in East Asia [26,30]. However, we cannot rule out the circulation of other genetic lineages of IDV (D/660, D/CA 2019, D/Yama 2019) in cattle farms in Punjab, Pakistan. A “One Health” approach is needed to unravel the complex interplay of phylodynamic factors, viral factors, host factors, and ecological factors to better monitor and control the spread of IDV. To date, the D/OK and D/660 lineages have been the most frequently reported lineages in multiple countries. The D/Yamagata/2016 and D/Yamagata/2019 lineages seem to be restricted to Japan and South Korea [29]. The United States and Australia are two main exporters of Holstein Friesian cattle to Pakistan. The circulation of the D/OK lineage in Pakistan could be linked to the import of cattle from these countries by private dairy companies. Further virological and serological studies, including whole genome sequencing, could help to validate our findings in this study. In addition, the circulation of D/OK lineage should be investigated in other animal species (sheep, goat, buffalo, camel, horses, deer). A multiple-sequence alignment comparison with reference strains (KT581418, KY441114) revealed some substitutions (Figures S1 and S2) in the nucleotide sequences of the IDV strains considered in the present study, which suggests that the D/OK lineage is undergoing evolutionary process which could potentially transmit to other species, including humans; however, further study is needed to elaborate this speculation. IDV strains with a reassortant genetic pattern have been identified, containing gene segments from both the D/OK and D/660 lineages [6]. Genetic analysis of IDV strains circulating in Europe from 2009 to 2022 showed increasing diversity due to mutations in the HEF glycoprotein (i.e., genetic drift), recombination, or the introduction of new clade strains. The nucleotide substitution rate of the IDV HEF glycoprotein was significantly higher than that of the ICV HEF [1,3,33,37].

IDV possess a zoonotic potential like influenza A viruses [38,39,40]. Recent studies detected IDV in nasal wash samples collected from swine farm workers in Malaysia [41], as well as in cattle farm workers [38]. In addition to this, IDV has been detected recently in airports [42] and hospitals [43] in the US. IDV could pose a serious zoonotic risk in the future owing to genetic reassortment and mutations. Therefore, animal farm workers need to remain vigilant and follow precautionary measures during farm operations. This study was limited by its small size in explaining the burden of IDV infection in Pakistan. Obtaining data on IDV carriage in dairy farms in Punjab or across Pakistan would have enhanced our understanding, but this was beyond the scope of this study. Similarly, serologic screening of these workers would have improved our understanding of IDV infection in this population. The sample collection process was challenging due to the lack of trained workers and restraint equipment on the dairy farm. These issues remain important areas of study for follow-up research.

In conclusion, this study confirms the presence of IDV for the first time in Pakistan. Our findings provide small-scale preliminary data about the epidemiology and genetic lineage of IDV. The detection of IDV indicates a potential health risk to cattle and farm workers. Therefore, it is prudent to conduct further surveillance studies throughout Pakistan to monitor seroprevalence, IDV burden, and its evolution in cattle and other animal species.

## Figures and Tables

**Table 1:** Animal species, location, sample data, and detection rate of influenza D virus in Punjab province, Pakistan.

Farm ID	Farm Location	Animal Type	Sex	Breed	Production Type	No of Animals	Age Range of Animals	Sample Type	No. Positive/No. Samples	Detection Rate, %	Accession Number	Lineage
A	Lahore	Cattle	Female	Holstein Friesian	Dairy	1300	2–6 years	Nasal swab	4/59 ^a^	6.8	PP763073, PP763074, PP763075, PP763076,	D/OK
B	Kasur	Cattle	Female	Holstein Friesian	Dairy	2000	2–6 years	Nasal swab	2/73 ^b^	2.7	PP763077, PP763078	D/OK
C	Sahiwal	Cattle	Female	Holstein Friesian	Dairy	1000	2–5 years	Nasal swab	0/49	0	Not detected	D/OK
D	Layyah	Cattle	Female	Cross bred	Dairy	155	2–6 years	Nasal swab	1/65 ^c^	1.5	PP763079	D/OK
E	Multan	Cattle	Female	Holstein Friesian	Dairy	1800	2–6 years	Nasal swab	2/77 ^d^	2.6	PP763080, PP763081	D/OK
F	Bahawalpur	Cattle	Female	Cross bred	Dairy	130	1–8 years	Nasal swab	0/53	0	Not detected	D/OK

^a^ Three IDV-positive cows were showing mild respiratory clinical signs (coughing, respiratory distress, respiratory sounds). ^b^ These IDV-positive cows were asymptomatic. ^c^ This IDV-positive cow was showing respiratory distress and diarrhea. ^d^ Both animals were showing mild respiratory distress. One had a recent history of mastitis while the other had a recent history of abortion.

## Data Availability

The data presented in this study are available upon request from the corresponding author. The data are not publicly available due to privacy or ethical restrictions.

## Supplementary Materials

The following supporting information can be downloaded at https://www.mdpi.com/article/10.3390/v16121865/s1: Figure S1. Multiple-sequence alignment comparison between the current IDV nucleotide sequences (496 bp) and reference sequences; Figure S2. Deduced amino acid comparison between IDV amino acid sequences (165 aa) of current study and reference sequences.
