# Supplementary material for: First Report of Influenza D Virus in Dairy Cattle in Pakistan
Source: Viruses. 2024 Nov 29;16(12):1865. doi: 10.3390/v16121865 (PMC11680143; doi:10.3390/v16121865)

Figure S1. Multiple sequence alignment results of the current IDV nucleotide sequences (496 bp) and corresponding published reference sequences.

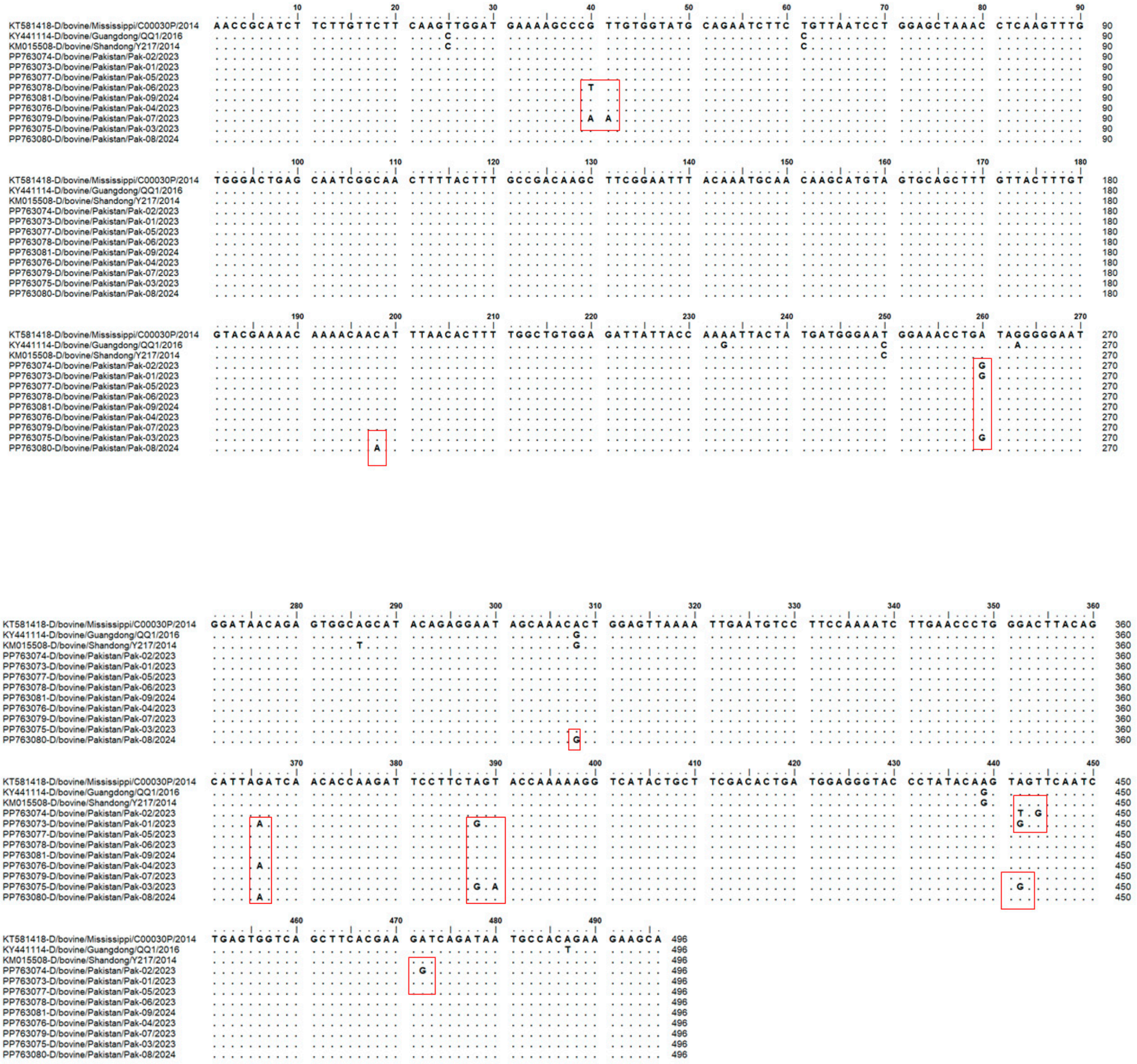

Figure S2. Multiple sequence alignment results of the current IDV amino acid sequences (165 aa) and corresponding published reference sequences.

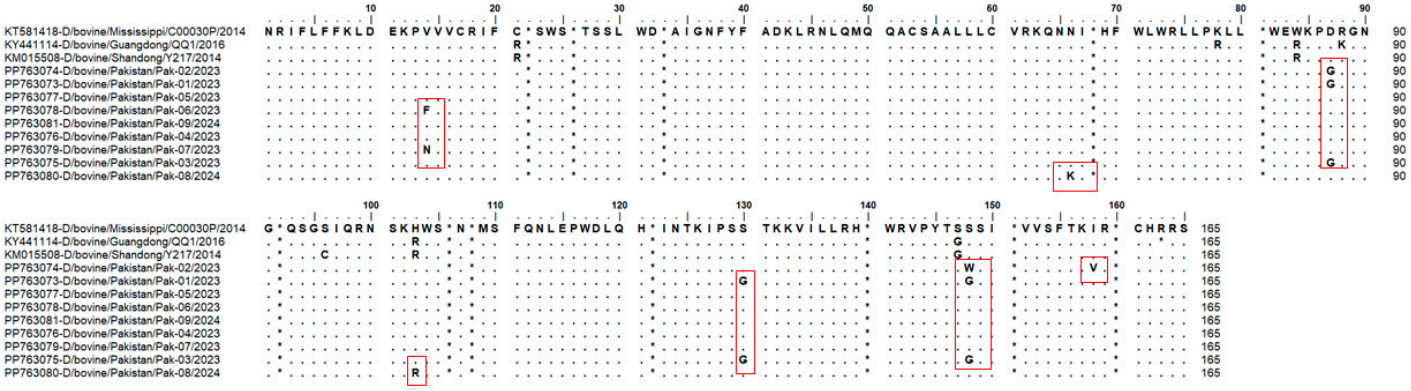

Supplement: Supplementary file 1 [file viruses-16-01865-s001.zip › viruses-3336717-supplementary.pdf]
